# Impact of 6% balanced hydroxyethyl starch following cardiopulmonary bypass on renal function: a retrospective study

**DOI:** 10.1186/s13019-020-01286-w

**Published:** 2020-09-07

**Authors:** Ju Yong Lim, Yun Seok Kim, Joon Bum Kim

**Affiliations:** 1 Department of Thoracic and Cardiovascular Surgery, Seoul St. Mary’s Hospital, College of Medicine, The Catholic University of Korea, Seoul, Republic of Korea; 2 Department of Thoracic and Cardiovascular Surgery, Keimyung University Dongsan Hospital, Daegu, Republic of Korea; 3 Department of Thoracic and Cardiovascular Surgery, Asan Medical Center, University of Ulsan College of Medicine, 88, Olympic-ro 43-gil, Songpa-gu, Seoul, 05505 Republic of Korea

**Keywords:** Hydroxyethyl starch, Acute kidney injury, Cardiopulmonary bypass

## Abstract

**Background:**

We aimed to evaluate the effect of limited volume of hydroxyethyl starch (HES) administration on postoperative renal function in patients undergoing cardiac surgery under cardiopulmonary bypass (CPB).

**Methods:**

One thousand six hundred fifty-seven patients undergoing cardiac surgery under CPB over two years were included. The patients were divided according to the amount of HES administrated during the first 2 days post-surgery; moderate dose HES (≥20 ml/kg) versus low dose HES (< 20 ml/kg). Outcomes were compared by using inverse probability weighting.

**Results:**

Incidence of acute kidney injury (AKI) was higher in the moderate HES group (*p* = .02). However, new renal replacement therapy (RRT) (*P* = .30) and early mortality (*p* = .97) was similar between the groups. When adjusted, the moderate HES use was associated with AKI (OR, 1.66; 95% CI, 1.12–2.44; *p* = .01), but did not increase the risk of new RRT (OR, 1.27; 95% CI, 0.71–2.18; *p* = .40) or early mortality (HR, 0.73; 95% CI, 0.29–1.81; *p* = .50).

**Conclusions:**

The moderate dose administration of HES (≥20 ml/kg) in the postoperative period following cardiac surgery might be associated with the risk of AKI. However, it was not associated with serious adverse outcomes such as new RRT or mortality. Further randomized controlled studies are needed to validate study results.

## Background

Synthetic colloids, hydroxyethyl starch (HES), have been widely used for perioperative fluid resuscitation in conjunction with crystalloids. With large molecular weight, HES is more effective volume expander than crystalloids with less pulmonary fluid accumulation and weight gain [1]. Thus, HES was used for pump priming and perioperative fluid therapy in patients undergoing cardiac surgery under cardiopulmonary bypass (CPB) who experienced increased capillary leakage in the immediate postoperative period. Despite of this beneficial aspect of HES, the toxic effects of HES are known to primarily involve the kidney by the accumulation of substitution of HES molecule in the kidney. Therefore, several randomized clinical trials [1–3] and meta-analysis [4] demonstrated the risks of HES administration outweigh the benefits compared to crystalloids in critically ill patients. Especially, as patients who undergo cardiac surgeries may be vulnerable to acute kidney injury (AKI) with nonpulsatility of flow and inflammatory response caused by CPB during surgery [5, 6], use of HES has been limited following cardiac surgery. In spite of the evolution of HES toward newer generations characterized by lower molecular weight and molar substitution to reduce renal toxicity, a recent study demonstrated it is also associated with a greater incidence of AKI in patients undergoing on-pump cardiac surgery [7]. Some suggested that less volume of HES administration would reduce adverse effect of HES [3, 8]. Therefore, HES was approved as a fluid therapy within limited its maximal volume: high molecular weight (MW) HES (670/0.75) up to 20 ml/kg/day and low MW HES (130/0.4) up to 50 ml/kg/day. However, safety issue in the use of limited volume of HES in patients after cardiovascular surgery is still controversial with conflicting study results. Some suggested that even moderate volume of HES administration more than 30 ml/kg/day might be associated with adverse effect of HES in the subset of cardiac surgery patients [9, 10]. Another study demonstrated that low volume of HES up to 20 ml/kg/day would be safe in the postoperative renal function following cardiac surgery using CPB. Therefore, with the lack of data and evidence of previous small cohort studies, we tried to evaluate the effect of limited volume of HES administration on postoperative renal function in patients undergoing cardiac surgery under CPB using data collected in the previous study for the impact of crystalloid administration on postoperative AKI [11].

## Methods

### Study population

**Fig. 1:**
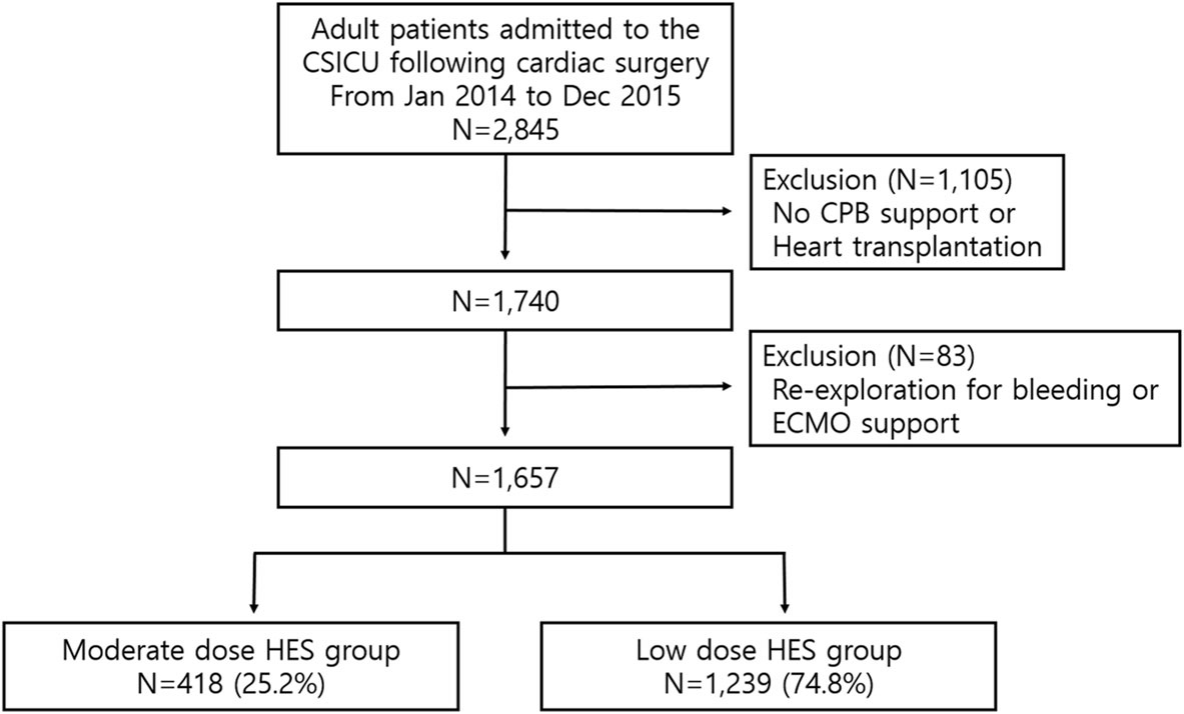
Study population

From January 2014 to December 2015, consecutive 2845 adult patients were admitted to the cardiac surgery ICU following cardiac surgery at our institution. Of these, we excluded patients undergoing cardiac surgery without CPB support or heart transplantation, and those receiving renal replacement therapy (RRT) preoperatively. Total of 1740 consecutive patients were enrolled and retrospectively reviewed. Among them, patients who underwent re-exploration for bleeding or extracorporeal membrane oxygenation support postoperatively were excluded to reduce potential confounding factors for AKI. Finally, 1657 patients were analyzed. To compare the effect of perioperative use of balanced HES on AKI, patients were grouped according to the amount of HES administrated during the first 2 days post-surgery; moderate dose HES (≥20 ml/kg) versus low dose HES (< 20 ml/kg) (Fig. 1). This study was approved and informed consent was waivered by the Institutional Review Board of our institution (IRB number:2016–0481).

### Intraoperative fluid management

In the operating room, the balanced buffered solution (Plasma Solution A, CJ Healthcare, Seoul, Korea) was used for all priming solution for CPB and additional volume requirements while running CPB. After discontinuation of CPB, fluid administration was performed to obtain proper preload based on the volume status on transesophageal echocardiography and other hemodynamic parameters such as central venous pressure, cardiac index, and stroke volume variation. A renal protective fluid strategy was followed by anesthesiologists; this consisted of a balanced crystalloid solution and limited amount of balanced HES (Hextend (670/0.75), CJ Healthcare, Seoul, Korea or Volulyte (130/0.4), Fresenius Kabi, Bad Homberg, Germany) [9]. Perioperative blood product transfusion in addition to fluid administration was performed according to the perioperative transfusion protocol of our institution.

### Postoperative fluid management

The initial fluid management strategy in our ICU included infusion of crystalloid solutions up to 1 L; 0.9% saline (JW Pharmaceutical, Seoul, Korea) and a balanced buffered solution, Plasma Solution A. If the crystalloid infusion was not effective as a volume expander and additional volume was required, 6% balanced HES solutions (Hextend or Volulyte) or blood products can be administered according to the patient’s renal function and coagulation. These decisions were made by the intensivist or the on-duty physician.

### Outcomes

The primary outcome was the incidence of postoperative AKI as defined by the Risk, Injury, Failure, Loss, End-stage classification and KDIGO definition (Kidney Disease: Improving Global Outcomes (KDIGO)). Secondary outcomes were the need for RRT and in-hospital mortality. The baseline creatinine level was based on the most recent available preoperative level prior to the operation. The postoperative creatinine level was measured daily during the first 2 days after surgery.

### Statistical analysis

Statistical analyses were performed using SAS version 9.3 (SAS Institute, Cary, NC). Data are expressed as mean ± standard deviation for continuous variables and as numbers and percentages for categorical variables. Data were examined for a normal distribution of variance with ANOVA and expressed as the mean ± SD. For an abnormal distribution of variance, data were assessed using a Kruskal-Wallis test and measured median (interquartile range). Preoperative and postoperative measurements were compared using Student’s paired-*t* test or the Mann–Whitney U test. The chi-square test or Fisher’s exact test was used to compare the categorical variables and to assess the statistical significance of differences between the two groups. A *p*-value of ≤ .05 was considered to be statistically significant in all comparisons. Univariate and multivariate analysis were performed for the entire patient cohort using logistic regression to determine predictors of early adverse outcomes. Variables were included in multivariate analysis if their univariate significance was < .10.

To reduce the impact of selection bias and potential confounding in an observational study, we also performed rigorous adjustment for baseline differences by use of the weighted logistic regression models with the inverse-probability-of treatment weighting (IPTW) [12]. With that technique, scores for patients receiving low dose HES (< 20 ml/kg) were weighted using the formula 1/(1-propensity score), whereas those for patients receiving moderate dose HES (≥20 ml/kg) were weighted using the formula 1/propensity score. The propensity scores, indicating the predicted probability of being moderate dose HES (≥20 ml/kg) conditional on the observed covariates, were estimated by multiple logistic-regression analysis. A full nonparsimonious model was developed, which included all the variables shown in Table 1. Model discrimination was assessed with c-statistics, and model calibration was assessed with Hosmer-Lemeshow statistics. The model was well calibrated (Hosmer-Lemeshow test; *p* = .89) with reasonable discrimination (c-statistics = 0.68).

**Table 1:** Baseline demographics and clinical characteristics

Variables	Low dose HES (*n* = 1239)	Moderate dose HES (*n* = 418)	*p*-value
Age	58.2 ± 13.6	59.4 ± 14.6	< .001
Female gender	503 (40.6)	251 (60.5)	.16
Diabetes mellitus	188 (15.9)	66 (15.7)	.76
Hypertension	514 (41.5)	163 (39.0)	.36
Cerebrovascular accident	65 (5.2)	18 (4.3)	.44
Euro score	2.6 ± 4.1	2.8 ± 4.8	.08
LV ejection fraction, %	58.7 ± 10.2	58.8 ± 10.5	.77
Medication			
Angiotensin receptor blockers	410 (33.1)	140 (33.5)	.88
Diuretics	483 (38.9)	177 (42.3)	.22
Insulin	121 (9.7)	35 (8.3)	.39
Statins	404 (32.6)	144 (34.4)	.48
Laboratory			
Hemoglobin, g/dL	12.9 ± 1.9	12.5 ± 1.8	< .001
Creatinine, mg/dL	0.89 (0.75, 1.06)	0.84 (0.69, 1.01)	< .001
Total bilirubin, mg/dL	0.5 (0.4, 0.8)	0.5 (0.4, 0.8)	.94

Results are presented as mean ± SD or median (IQR) as appropriate and number (percentage)

*HES* Hydroxyethyl starch; *LV* Left ventricle

## Results

Of the 1657 patients enrolled, 418 (25.2%) were included in the moderate dose HES group and 1239 (74.8%) in the low dose HES group. The baseline characteristics of the patients in both groups are summarized in Table 1. More female patients were included in the moderate dose HES group (*p* < .001) and CPB time was longer in the moderate dose HES group (*p* = .02). Perioperatively, more red blood cells and fresh frozen plasma were transfused in moderate dose HES group (*p* < .05). The incidence rate of AKI was significantly higher in moderate dose HES group in the univariate and multivariate analysis (OR, 1.80; 95% CI, 1.22–2.64; *p* = .003, and OR, 1.72; 95% CI, 1.15–2.58; *p* = .008). However, the need for new RRT were similar between the groups. These perioperative findings and clinical outcomes are summarized in Tables 2,3.

**Table 2:** Perioperative findings of low dose HES vs moderate dose HES group

Variables	Low dose HES (n = 1239)	Moderate dose HES (n = 418)	*p*-value
CPB time, min	142.5 ± 65.1	150.8 ± 65.1	.02
ACC time, min	92.4 ± 48.0	97.9 ± 48.4	.05
Operation			.23
Valve	789 (63.6)	279 (66.5)	
CABG	88 (7.1)	20 (4.7)	
Valve + CABG	43 (3.4)	15 (3.5)	
Aorta	171 (13.8)	65 (15.5)	
Others	148 (11.9)	39 (9.3)	
Crystalloid administration			
Saline, L	0.9 ± 1.0	0.8 ± 0.2	.76
Balanced, L	3.1 ± 0.7	3.0 ± 0.8	.68
6% HES			
670/0.75, L	0.21 ± 0.2	0.8 ± 0.3	< .001
670/0.75, ml/kg	3.9 ± 4.2	14.5 ± 4.9	< .001
130/0.4, L	0.4 ± 0.1	0.6 ± 0.2	< .001
130/0.4, ml/kg	7.8 ± 2.4	11.4 ± 5.3	< .001
Transfusion			
Red blood cell, L	0.9 ± 1.7	1.1 ± 1.6	.009
Fresh frozen plasma, L	0.6 ± 1.4	0.6 ± 1.4	.24
Cryoprecipitate, L	0.9 ± 1.9	1.3 ± 2.0	< .001
Platelet concentrate, L	0.9 ± 2.5	1.0 ± 2.9	.15

Results are presented as mean ± standard deviation or number (percentage). *HES* Hydroxyethyl starch; *CPB* Cardiopulmonary bypass; *ACC* Aorta cross-clamp; *CABG* Coronary artery bypass graft

**Table 3:** Clinical outcomes of low dose HES vs moderate dose HES group

Variables	Low dose HES (*n* = 1239)	Moderate dose HES (*n* = 418)	*p*-value
Acute kidney injury	78 (6.3)	45 (10.7)	.02
RIFLE (Risk)	56 (4.5)	34 (8.1)	
RIFLE (Injury)	18 (1.4)	8 (1.9)	
RIFLE (Failure)	4 (0.3)	3 (0.7)	
KDIGO	101 (8.2)	57 (13.6)	.02
New renal replacement therapy	40 (3.2)	18 (4.3)	.30
ICU stay, days	2.9 ± 7.7	2.9 ± 2.8	.003
30-day mortality	21 (1.6)	7 (1.6)	.97
Mortality after 30-day	37 (2.9)	22 (5.2)	.03

Results are presented as mean ± standard deviation or number (percentage). *HES* Hydroxyethyl starch; *RIFLE* Risk, Injury, Failure, Loss of kidney function, End-stage kidney disease classification to define and stratify the severity of acute kidney injury; *KDIGO* Kidney disease improving global outcomes; *ICU* Intensive care unit

### Risk adjusted outcomes

After adjustment with IPTW, requirement of new RRT after surgery was similar between groups (OR, 1.27; 95% CI, 0.71–2.18; *p* = .40). However, this identified increased risk of AKI in moderate dose HES group by RIFLE classification (OR, 1.66; 95% CI, 1.12–2.44; *p* = .01), and KDIGO definition (OR, 1.68, 95% CI, 1.18–2.36; *p* = .003). In-hospital mortality was not associated with moderate dose HES group (HR, 0.73; 95% CI, 0.29–1.81; *p* = .50).

**Fig. 2:**
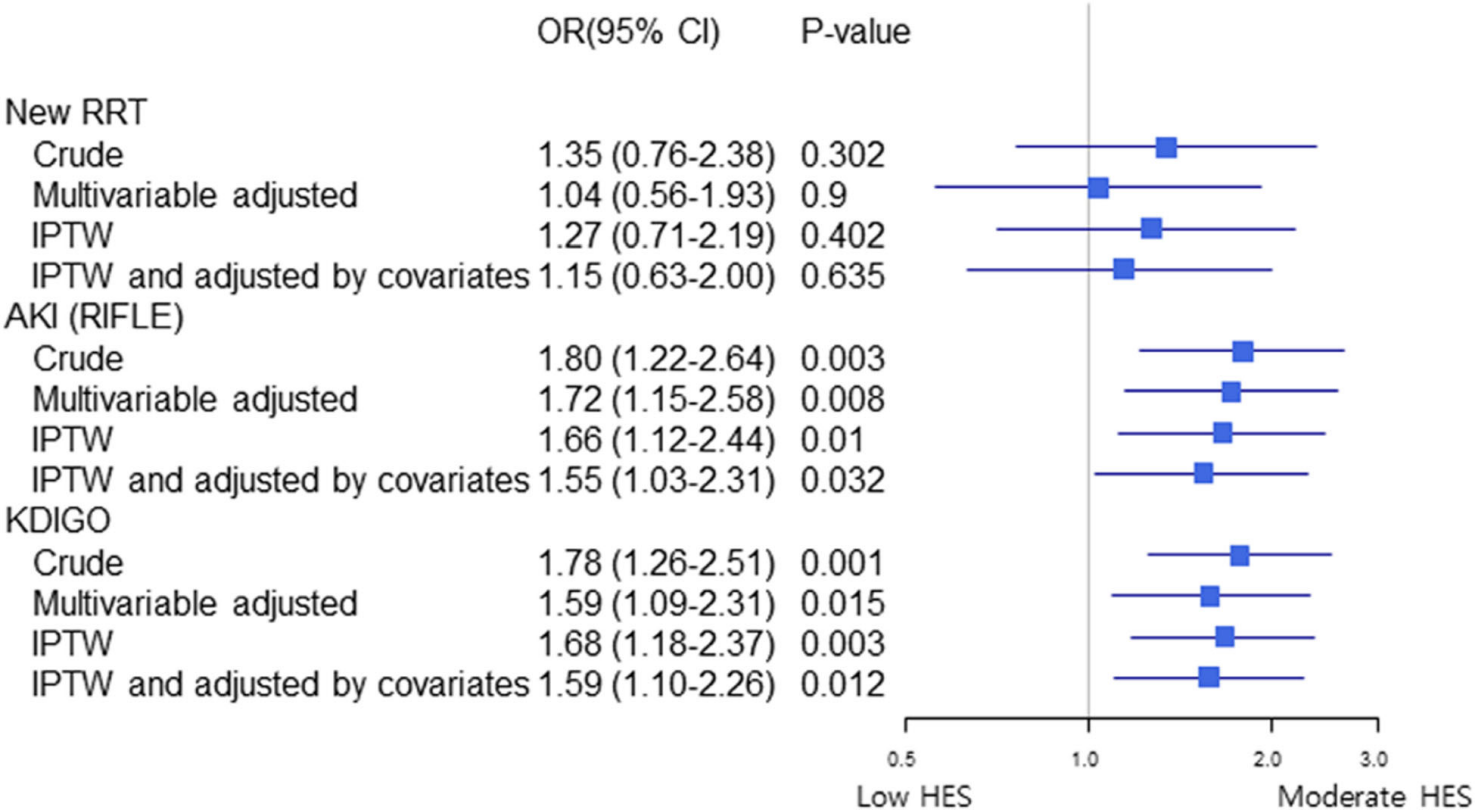
Forest plots for outcomes. OR, Odds ratio; CI, Confidence interval; RRT, renal replacement therapy; RIFLE, Risk, Injury, Failure, Loss of kidney function, End-stage kidney disease classification to define and stratify the severity of acute kidney injury; KDIGO, Kidney Disease: Improving Global Outcomes; IPTW, Inverse probability of treatment weighting; HES, hydroxyethyl starch

The two clinically significant covariates (the use of fresh frozen plasma, cryoprecipitate transfusion) were adjusted in combination with IPTW. This analysis also demonstrated that perioperative moderate dose HES administration affect AKI (OR, 1.55; 95% CI, 1.03–2.31; *p* = .03) but not on requirement of new RRT and in-hospital mortality (OR, 1.14; 95% CI, 0.63–2.00; *p* = .63, and HR, 1.72; 95% CI, 0.75–3.96; *p* = .20, respectively). This analysis of each outcomes was summarized in Fig. 2.

## Discussion

This study compared the impact of moderate dose and low dose HES administration on kidney function after cardiac surgery using CPB. We found that moderate dose HES administration was associated with increased risk of postoperative AKI development, but not with the risk of the need for new RRT or in-hospital mortality. These findings remained consistent after adjustment of the baseline characteristics with IPTW model.

According to the systemic review by Mutter et al. [13], effects of HES on RRT or AKI defined by RIFLE criteria were reviewed compared to other fluid therapy. The results suggested that either high (≥2 L) or low (< 2 L) volume of HES increased the risk of new RRT. However, regarding AKI defined by RIFLE criteria, volume of HES was not associated with increasing the risk. However, these patients included in this review were mostly septic or non-septic patients which might not be comparable to those who undergo cardiac surgery under CPB. Pathophysiology of kidney injury by CPB are not clear, but CPB was known to attribute to cellular ischemia and consequent injuries to tubular and vascular endothelium of the kidney [14–17]. Also, low mean arterial pressure and nonpulsatile flow during CPB lead to impair kidney autoregulation [14], which may make cardiac surgery patients vulnerable to AKI postoperatively. A recent study demonstrated that intraoperative use of HES is associated with a greater incidence of AKI in patients undergoing on-pump cardiac surgery [7]. However, other prospective observational study of colloids in a large cohort study by Ryhammer et al. [18] insisted that using HES in cardiac surgery patients were not related to new RRT or early or late mortality. This suggested that harmful effect of HES might not be serious than expected. Nevertheless, with the caution of renal toxicity, an effort was made to minimize the potential harmful effect on renal function of HES by limiting the volume administrated after off-pump coronary artery bypass grafting surgeries [9]. We also tried to restrict to administrate HES in addition to the first line fluid therapy with crystalloid in postoperative management after cardiac surgery in conjunction with renal protective strategy of anesthesiology in our hospital. Maximal volume of moderate dose HES group was 44 ml/kg for 2 days, which was less than the approved maximal volume in our country. We assume that the volume of HES administrated in moderate dose HES group was also restricted, so there was no intergroup difference regarding the risk of new RRT or early mortality even though it seemed associated with AKI diagnosed with increasing level of serum creatinine. Rather, transfusions such as fresh frozen plasma, cryoprecipitates were risk factors for severe AKI, new RRT, or early mortality.

Furthermore, in the immediate postoperative period when capillary leakage increases because of various inflammatory response caused by CPB, HES may be more effective volume expander than crystalloids with less pulmonary fluid accumulation and weight gain [1]. As shown in our study result, if the renal toxic effect of moderate dose HES are not severe, and low dose HES could be used with relatively safe, benefits and risks should be weighed in the postoperative management setting after cardiac surgery.

There were several limitations to our study. First, this was a retrospective observational study and was not a randomized controlled trial. Comparison between the two groups may be biased by potential confounding factors even though we performed a propensity weighting and multivariate adjustment analysis to reduce any potential bias. Second, definition of moderate dose of HES (≥ 20 ml/Kg) might be artificial with lack of evidence. Therefore, the amount of HES administrated in the moderate dose HES group might not be enough to develop severe renal dysfunction. Third, two types of HES with different molecular weights and molar substrates were used in our study. This difference between the products which might have contributed to AKI was not considered and could act as a confounding factor in addition to other operative and postoperative factors.

## Conclusions

The moderate dose administration of HES (≥20 ml/kg) in the postoperative period following cardiac surgery might be associated with the risk of AKI. However, it was not associated with severe renal dysfunction including requirement of new RRT. Also, it did not increase the mortality rate. Benefits and risks should be weighed in the administration of HES. Further randomized controlled studies are needed to validate study results.

## Data Availability

The datasets used and analyzed during the current study are available from the corresponding author on reasonable request.

## Abbreviations

AKI: Acute kidney injury
CPB: Cardiopulmonary bypass
HES: Hydroxyethyl starch
ICU: Intensive care unit
IPTW: Inverse-probability-of treatment weighting
KDIGO: Kidney disease: improving global outcomes
MW: Molecular weight
RIFLE: Risk, injury, failure, loss, end-stage kidney disease
RRT: Renal replacement therapy
